# Predictive factors for the presence and long-term persistence of SARS-CoV-2 antibodies in healthcare and university workers

**DOI:** 10.1038/s41598-022-13450-4

**Published:** 2022-06-13

**Authors:** Céline Grégoire, Pascale Huynen, Stéphanie Gofflot, Laurence Seidel, Nathalie Maes, Laura Vranken, Sandra Delcour, Michel Moutschen, Marie-Pierre Hayette, Philippe Kolh, Pierrette Melin, Yves Beguin

**Affiliations:** 1 Division of Hematology, CHU Sart-Tilman, Avenue de l’hôpital 1, 4000 Liège, Belgium; 2 Division of Medical Microbiology, Unilab, CHU of Liège, Liège, Belgium; 3 Center for Interdisciplinary Research On Medicines, University of Liège, Liège, Belgium; 4 Biothèque Hospitalo-Universitaire de Liège (BHUL), CHU of Liège, Liège, Belgium; 5 Department of Biostatistics and Medico-Economic Information, CHU of Liège, Liège, Belgium; 6 Unilab, CHU of Liège, Liège, Belgium; 7 Division of Infectious Diseases and General Internal Medicine, CHU of Liège, Liège, Belgium; 8 GIGA-I3 Laboratory of Immunology, University of Liège, Liège, Belgium; 9 Department of Information System Management, CHU of Liège, Liège, Belgium; 10 Department of Biomedical and Preclinical Sciences, University of Liège, Liège, Belgium; 11 GIGA-I3 Laboratory of Hematology, University of Liège, Liège, Belgium

**Keywords:** Viral infection, Viral infection, Humoral immunity, Risk factors, Occupational health

## Abstract

While patient groups at risk for severe COVID-19 infections are now well identified, the risk factors associated with SARS-CoV-2 (severe acute respiratory syndrome coronavirus 2) transmission and immunization are still poorly understood. In a cohort of staff members of a Belgian tertiary academic hospital tested for SARS-CoV-2 antibodies during the early phase of the pandemic and followed-up after 6 weeks, 3 months and 10 months, we collected personal, occupational and medical data, as well as symptoms based on which we constructed a COVID-19 score. Seroprevalence was higher among participants in contact with patients or with COVID-19 confirmed subjects or, to a lesser extent, among those handling respiratory specimens, as well as among participants reporting an immunodeficiency or a previous or active hematological malignancy, and correlated with several symptoms. In multivariate analysis, variables associated with seropositivity were: contact with COVID-19 patients, immunodeficiency, previous or active hematological malignancy, anosmia, cough, nasal symptoms, myalgia, and fever. At 10 months, participants in contact with patients and those with higher initial COVID-19 scores were more likely to have sustained antibodies, whereas those with solid tumors or taking chronic medications were at higher risk to become seronegative.

## Introduction

Since the beginning of the COVID-19 (COronaVIrus Disease 2019) pandemic, virtually no one has been spared exposure to SARS-CoV-2 (Severe Acute Respiratory Syndrome CoronaVirus 2). Numerous epidemiological studies have been conducted in the general population or among healthcare workers, but few data are available on subject characteristics, direct exposure, and symptoms in large cohorts. Using data from our four-phase monitoring of the humoral response to SARS-CoV-2 infection in nearly 4000 volunteer healthcare and non-healthcare workers^1^, we aimed to identify predictors of SARS-CoV-2 seropositivity and long-term persistence of these antibodies. While we used several serological tests to identify IgM, IgA and IgG in the principal study, we focused on the results of the IgG DiaSorin assay for this part of the study. SARS-CoV-2 serologic tests were performed on days 0, 45, and 90, and participants with positive results in phases 1, 2, and/or 3 (positive for ≥ 1 IgG and/or for ≥ 2 IgM or IgA tests) were again invited to undergo SARS-CoV-2 IgG testing 10 months after the first sampling.

## Results

### Phase 1

Between April 6 and May 5 2020, 3776 staff members were tested (74.7% women, median age 39.8 years), and 336 (8.9%) participants had a detectable IgG immune response against SARS-CoV-2 using the DiaSorin assay. As expected, IgG seroprevalence significantly increased over time (time calculated from March 1, date of the first Belgian case of COVID-19, in weeks; OR 1.044 [95% CI 1.028–1.060], *p* < 0.0001), from 6.3% among participants tested during the first week (week 5 of the pandemic in Belgium) to 14.0% in participants tested during the last week of phase 1 (week 8 of the pandemic in Belgium). In subsequent analyses, all other variables were therefore adjusted for time between March 1 and testing.

Among the 3776 participants, 3719 consented to share personal and health data and were included in the subsequent analyses. Seroprevalence was similar regardless of gender, age, living area or recent travels, and tended to increase with weight (although this did not reach statistical significance; OR 1.007 [95% CI 0.999–1.015], *p* = 0.078), but not with body mass index. IgG seropositivity was not different in smokers and non smokers, but tended to decrease, although not significantly, with the number of cigarettes smoked per day (OR 0.941 [95% CI 0.884–1.002], *p* = 0.059) (Table 1). SARS-CoV-2 seropositivity was significantly higher in participants with previous or active hematological malignancy (31.3% vs 8.7%, OR 4.83 [95% CI 1.65–14.14], *p* = 0.004) and in those with an immunodeficient status (26.3% vs 8.7%, OR 3.28 [95% CI 1.16–9.26], *p* = 0.02). There was no impact of other severe comorbidities, ongoing pregnancy, or chronic medications (Supplementary Table S1).

**Table 1:** Predictors of SARS-Cov-2 positive serology (IgG DiaSorin) in phase1: subject characteristics.

Predictive factor		*N* (%)	Mean ± SD (range)	Prevalence	OR (95% CI)	*P* value
Overall		3776	–	336 (8.9%)	–	–
Sex	Female	2821 (74.7%)	–	247 (8.8%)	–	
	Male	955 (25.3%)	–	89 (9.3%)	1.08 (0.84–1.40)	0.55
Age	(Year)	3776	41.0 ± 11.7 (20.1–81.3)	–	0.992 (0.982–1.002)	0.102
Weight	(Kilogram)	3719^a^	70.1 ± 14.2 (33–150)	–	1.007 (0.999–1.015)	0.078
Height	(Centimeter)	3719^a^	169.0 ± 8.9 (125–200)	–	1.010 (0.998–1.023)	0.11
BMI	(kg/m^2^)	3719^a^	24.5 ± 4.3 (15.6–55.1)	–	1.015 (0.990–1.042)	0.24
Smoking	No	3219^a^ (86.6%)	–	291 (9.0%)	–	
	Yes	500^a^ (13.4%)	–	37 (7.4%)	0.76 (0.53–1.09)	0.14
Tobacco consumption	(Cigarette/day)	471^b^	9.96 ± 6.34 (0.05–40)	–	0.941 (0.884–1.002)	0.059

Place of residence, specific workplace, number/country/period of travel abroad, and type of mask used were also tested and found not significant. SD: standard deviation; BMI = body mass index; OR = odds ratio of univariate logistic regression models adjusted for time between March 1 and testing; CI = confidence interval.

^a^57 subjects did not consent to sharing personal health data.

^b^29 subjects who reported being smokers did not share their tobacco consumption.

Regarding risk of exposure, we observed a higher seroprevalence in staff members working in contact with patients (9.5% vs 7.5%, OR 1.30 [95% CI 1.01–1.68], *p* = 0.04), and particularly in nurses (10.6%; OR 1.57 [95% CI 1.06–2.33] compared to the administrative staff) (Table 2). Additional detailed comparisons (27 categories) were performed: compared to secretaries (who are not in contact with patients), seroprevalence was higher in most occupational categories in direct contact with patients (reception staff members, nurse assistants, porters, occupational therapists, nurses, physicians and physicians in training). The other functions were not identified as risk factors (Supplementary Table S2). Participants with at least one contact with a COVID-19 subject had a higher seroprevalence (10.8% vs 7.3%, OR 1.51 [95% CI 1.20–1.89], *p* = 0.0004), with the highest prevalence observed in case of household contact (25.4%; OR 4.12 [95% CI 2.27–7.48]), followed by frequent (>3 in total) or daily contacts with COVID-19 patients at work (11.0%; OR 1.53 [95% CI 1.18–2.00]) (Table 2). Laboratory workers handling respiratory specimens tended to have a higher SARS-CoV-2 seroprevalence (13.5% vs 8.8%, OR 1.82 [95% CI 0.97–3.39], *p* = 0.062). There was no significant difference according to type of mask used (if any), but this differed strongly with exposure (indeed, at the beginning of the pandemic, mask availability was insufficient—particularly for FFP2 masks, and their use was therefore adapted to the risk of exposure).

**Table 2:** Predictors of SARS-Cov-2 positive serology (IgG DiaSorin) in phase 1: direct exposure.

Predictive factor		*N* (%)	Prevalence	OR (95% CI)	*P* value
Time	Weeks from March 1	3776	–	1.044 (1.028–1.060)	< 0.0001
Function	Administrative staff	477 (12.6%)	34 (7.1%)	–	0.041
	Researcher	235 (6.2%)	20 (8.5%)	1.19 (0.67–2.13)	
	Laboratory staff	228 (6.0%)	19 (8.3%)	1.28 (0.71–2.31)	
	Technical staff	430 (11.4%)	30 (7.0%)	0.89 (0.54–1.49)	
	Paramedical staff	461 (12.2%)	34 (7.4%)	0.97 (0.59–1.60)	
	Nurse	1233 (32.7%)	131 (10.6%)	1.57 (1.06–2.33)	
	Physician	712 (18.9%)	68 (9.6%)	1.41 (0.91–2.16)	
Patient contact	No	1156 (30.6%)	87 (7.5%)	–	
	Yes	2620 (69.4%)	249 (9.5%)	1.30 (1.01–1.68)	0.043
Handling of respiratory specimens	No	3687 (97.6%)	324 (8.8%)	–	
	Yes	89 (2.4%)	12 (13.5%)	1.82 (0.97–3.39)	0.062
Contact with COVID patients	No	2056 (54.4%)	151 (7.3%)	–	
	Yes	1720 (45.6%)	185 (10.8%)	1.51 (1.20–1.89)	0.0004
Type of contact	None	2056 (54.4%)	151 (7.3%)	–	< 0.0001
	Familial	63 (1.7%)	16 (25.4%)	4.12 (2.27–7.48)	
	Occasional at work	711 (18.8%)	65 (9.1%)	1.27 (0.94–1.73)	
	Frequent/daily at work	946 (25.1%)	104 (11.0%)	1.53 (1.18–2.00)	
COVID infection	No	3632 (96.2%)	253 (7.0%)	–	
	Yes	144 (3.8%)	83 (57.6%)	16.16 (11.3–23.2)	< 0.0001
COVID diagnosis	No infection	3632 (96.2%)	253 (7.0%)	–	< 0.0001
	PCR diagnosis	82 (2.2%)	61 (74.4%)	34.24 (20.4–57.5)	
	Clinical diagnosis	62 (1.6%)	22 (35.5%)	6.69 (3.90–11.48)	

OR = odds ratio of univariate logistic regression models adjusted for time between March 1 and testing; CI = confidence interval.

In our population, 144 individuals (3.8%) had been diagnosed with COVID-19, either by RT-PCR or by clinical evaluation (diagnosis made by the general practitioner on the basis of symptoms, without performing a confirmatory RT-PCR test), and this was associated with a higher SARS-CoV-2 seroprevalence (57.6% vs 7.0%, OR 16.2 [95% CI 11.3–23.2], *p* < 0.0001). Whereas COVID-19 diagnosed by PCR resulted in a seropositivity rate of 74.4%, clinical diagnosis without PCR performed was already associated with a seropositivity rate of 35.5% (Table 2). Symptoms consistent with COVID-19 in preceding weeks were reported by 600 individuals, which was significantly associated with seroprevalence, globally (25.3% vs 5.8%, OR 5.46 [95% CI 4.30–6.92], *p* < 0.0001) or for each symptom individually. Our 17-point COVID-19 score based on these symptoms also correlated with seroprevalence (OR 1.25 [95% CI 1.22–1.29], *p* < 0.0001) (Table 3).

**Table 3:** Predictors of SARS-Cov-2 positive serology (IgG DiaSorin) in phase 1: symptoms.

Predictive factor		*N* (%)	Prevalence	OR (95% CI)	*P* value
Symptoms before testing	No	3176 (84.1%)	184 (5.8%)	–	
	Yes	600 (15.9%)	152 (25.3%)	5.46 (4.30–6.92)	< 0.0001
Covid score^a^	1–3	186 (4.9%)	16 (8.6%)	1.62 (0.95–2.76)	< 0.0001
	4–10	313 (8.3%)	86 (27.5%)	6.03 (4.51–8.07)	
	11–17	101 (2.7%)	50 (49.5%)	14.4 (9.46–22.0)	
Cough	No	3516 (93.1%)	258 (7.3%)	–	
	Yes	260 (6.9%)	78 (30.0%)	5.24 (3.90–7.05)	< 0.0001
Dyspnea	No	3671 (97.2%)	303 (8.3%)	–	
	Yes	105 (2.8%)	33 (31.4%)	4.82 (3.12–7.43)	< 0.0001
Anosmia	No	3665 (97.1%)	261 (7.1%)	–	
	Yes	111 (2.9%)	75 (67.6%)	24.6 (16.1–37.4)	< 0.0001
Ageusia	No	3665 (97.1%)	271 (7.4%)	–	
	Yes	111 (2.9%)	65 (58.6%)	16.0 (10.7–23.9)	< 0.0001
Nasal symptoms	No	3530 (93.5%)	272 (7.7%)	–	
	Yes	246 (6.5%)	64 (26.0%)	4.19 (3.06–5.74)	< 0.0001
Sore throat	No	3577 (94.7%)	302 (8.4%)	–	
	Yes	199 (5.3%)	34 (17.1%)	2.19 (1.48–3.23)	< 0.0001
Abdominal pain	No	3691 (97.7%)	316 (8.6%)	–	
	Yes	85 (2.3%)	20 (23.5%)	2.99 (1.78–5.02)	< 0.0001
Diarrhea	No	3644 (96.5%)	297 (8.2%)	–	
	Yes	132 (3.5%)	39 (29.6%)	4.45 (2.99–6.62)	< 0.0001
Vomiting	No	3763 (99.7%)	331 (8.8%)	–	
	Yes	13 (0.3%)	5 (38.5%)	5.82 (1.87–18.1)	0.0024
Myalgia	No	3545 (93.9%)	251 (7.1%)	–	
	Yes	231 (6.1%)	85 (36.8%)	7.23 (5.36–9.76)	< 0.0001
Headaches	No	3443 (91.2%)	239 (6.9%)	–	
	Yes	333 (8.8%)	97 (29.1%)	5.39 (4.10–7.08)	< 0.0001
Fever	No	3632 (96.2%)	274 (7.5%)	–	
	Yes	144 (3.8%)	62 (43.1%)	8.44 (5.91–12.04)	< 0.0001

OR = odds ratio of univariate logistic regression models adjusted for time between March 1 and testing; CI = confidence interval.

^a^COVID-19 score: cough or dyspnea = 4 points; anosmia or ageusia = 4 points; nasal symptoms or sore throat = 1 point; abdominal pain, diarrhea or vomiting = 1 point; myalgia = 1 point; headaches = 2 points; fever < 38 °C = 1 point or fever ≥ 38 °C = 4 points.

**Figure 1:**
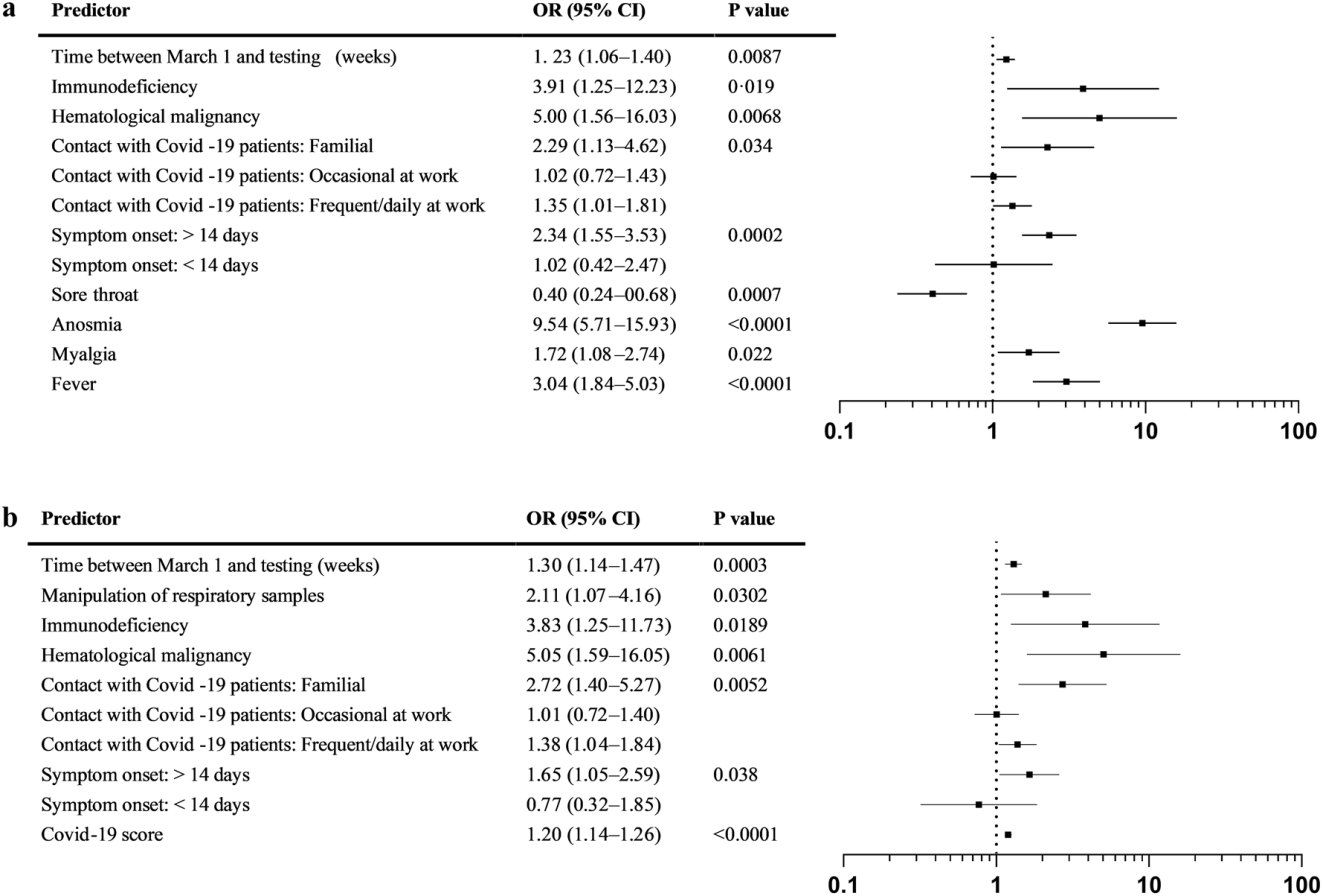
Predictors of SARS-Cov-2 positive serology (IgG DiaSorin) in phase 1 in multivariate analyses. (**a**) Binary model with subject characteristics, exposure and detailed symptoms. (**b**) Binary model with subject characteristics, exposure and COVID-19 score.

Multivariate analyses included all variables with *p*-values < 0.10 in univariate analyses (excluding the previous diagnosis of COVID-19), with symptoms either detailed separately or pooled in our COVID-19 score. In the first model including all symptoms, significant variables associated with higher SARS-CoV-2 seroprevalence were: time between March 1 and testing, immunodeficiency, previous or active hematological malignancy, type of contact with COVID-19 patients, former (> vs < 14 days) symptoms, three previous symptoms i.e. anosmia, myalgia and fever, while sore throat was associated with a lower seroprevalence (Fig. 1a). In the second model where individual symptoms were replaced by the COVID-19 score, the latter was significantly associated with a higher seroprevalence, as well as the same variables as in the first model, with the addition of handling of respiratory specimens (Fig. 1b).

### Phases 2 and 3

When analyzing the evolution of IgG after 6 (phase 2) and 12 weeks (phase 3) in 3187 and 2498 subjects who completed phases 2 and 3, respectively, we observed broadly the same variables associated with seroprevalence overall in phases 1–2–3 in univariate analyses adjusted for time between March 1 and testing, except for the appearance of a negative correlation between seroprevalence and age (OR 0.989 [95% CI 0.980–0.998], *p* = 0.013) (Supplementary Tables S3–S6). In multivariate analysis, in the first model including all symptoms, significant variables associated with higher seroprevalence overall in phases 1–2–3 were: type of contact with COVID-19 patients, immunodeficiency, previous or active hematological malignancy, five previous symptoms i.e. anosmia, myalgia, cough, nasal symptoms and fever, while sore throat and abdominal pain were associated with lower seroprevalence. In the second model in which individual symptoms were replaced by the COVID-19 score, significant variables associated with a higher seroprevalence were time between March 1 and testing, type of contact with COVID-19 patients, immunodeficiency, previous or active hematological malignancy, and COVID-19 score, while variables associated with lower seroprevalence were age and smoking (Supplementary Fig. S1a-b).

### Phase 4

**Figure 2:**
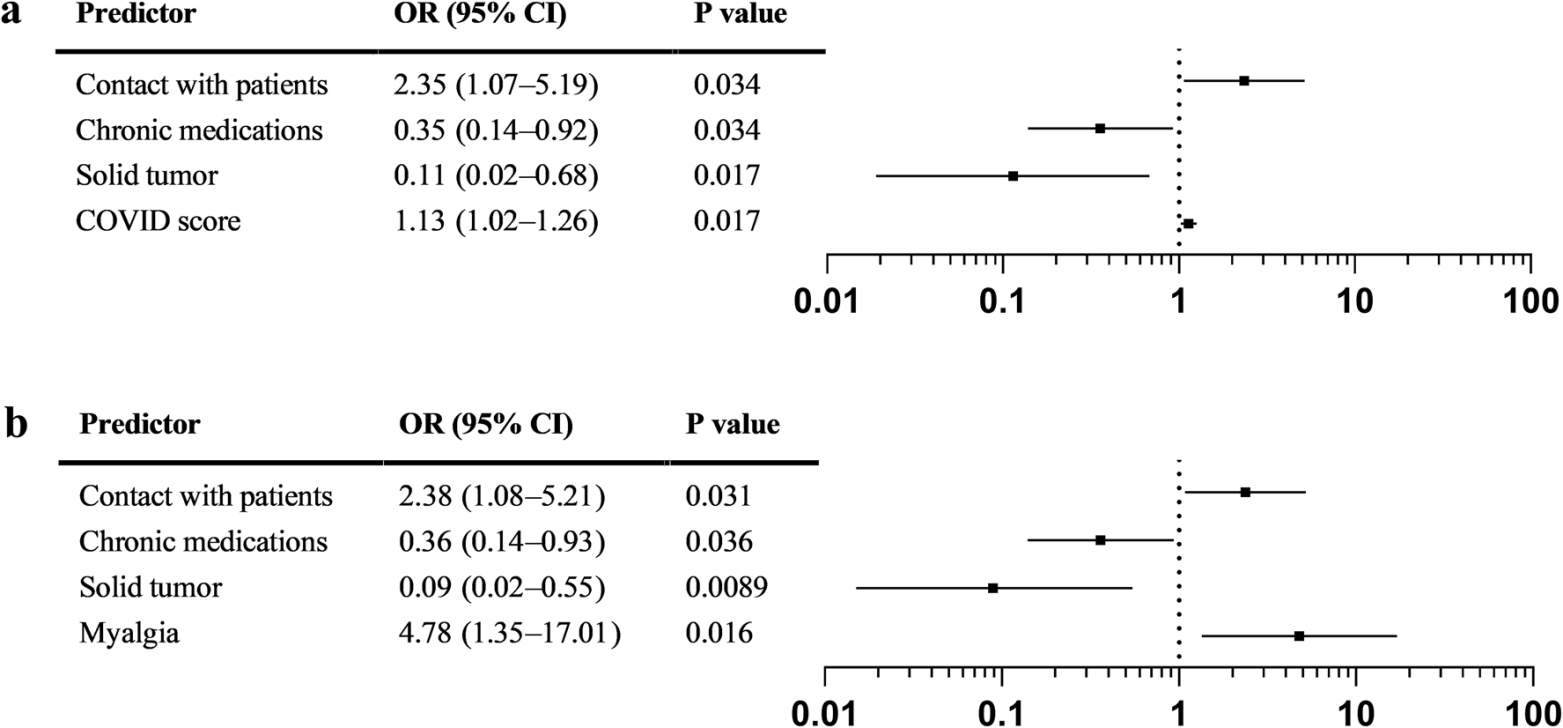
Predictors of persistent SARS-Cov-2 positive serology (IgG DiaSorin) in phase 4 in multivariate analyses. (**a**) Binary model with subject characteristics, exposure and COVID-19 score. (**b**) Binary model with subject characteristics, exposure and detailed symptoms.

Ten months later, 277 (88.5%) of 313 previously seropositive participants had detectable IgG by the DiaSorin test. Factors associated with sustained seropositivity in both univariate and multivariate analyses were contact with patients and most symptoms (only myalgia in the multivariate analysis) as well as our COVID-19 score, whereas participants with a solid tumor or taking any chronic medication were more likely to become seronegative (Fig. 2a, b).

## Discussion

In our cohort, analysis of personal and occupational factors of our participants identified several predictors of SARS-CoV-2 IgG seropositivity. As expected at the beginning of a pandemic, seroprevalence increased with time throughout the study weeks, and was higher among workers in contact with patients, but also, to a lesser extent, among laboratory staff handling respiratory specimens. Contact with COVID-19 patients increased the risk of seroconversion, with a higher risk for household contacts than for contacts with infected patients or colleagues (as confirmed in other cohorts^2^). These results highlight the value of protective equipment at work, and should encourage us to maintain strict measures in hospitals, and even to reinforce protective protocols for all healthcare workers in contact with patients, and particularly with COVID-19 patients (or with respiratory samples from them).

We confirmed the pertinence of the COVID-19 score used to screen patients at risk for SARS-CoV-2 infection. Anosmia, fever, myalgia and cough were the strongest predictive symptoms of seropositivity. Importantly, 5.8% of asymptomatic participants had a positive serology, emphasizing the need for large-scale screening strategies to detect asymptomatic carriers as part of a risk management strategy. On the other hand, 21/82 participants diagnosed with COVID-19 based on RT-PCR had a negative serology, probably because most of them experienced a mild form of the disease (only one subject from our cohort was hospitalized). Indeed, antibody levels were correlated with symptom severity in several studies^3–5^, which was confirmed in our cohort. In those patients who did not develop detectable anti-SARS-CoV-2 antibodies despite PCR-proven infection, a certain degree of immune response is nevertheless possible as cellular immune responses (not evaluated here) also participate to SARS-CoV-2 immunity^6^.

When analyzing the serological evolution after 10 months, being in contact with patients and a higher COVID-19 score were associated with persistent anti-Sars-CoV2 IgG. Recurrent exposure to SARS-CoV-2 could possibly have contributed to stability of IgG levels in some healthcare workers in contact with patients. Indeed, several studies have shown the persistence of IgG^+^ memory B cells after SARS-CoV-2 infection, which suggests the possibility of a rapid antibody response upon re-exposure^7,8^.

Participants reporting an immunocompromised status or a previous or active hematological malignancy (but not a solid tumor) had a higher seroprevalence. This could appear contradictory to other studies that have shown an impaired humoral response to natural infection with SARS-CoV-2 in patients with hematological malignancies, and to a lower extent in those with solid tumors^9^. However, in contrast to these studies, most of the subjects in our cohort who reported a history of hematological malignancy (mostly lymphoma) were young, in remission and without treatment. Weaker humoral responses to SARS-CoV-2 vaccines have also been described in patients suffering from hematological malignancies, but mostly in patients undergoing treatment or recently treated with anti-B-cell therapies, or after allogeneic transplantation^10–13^. There are now consistent data showing that immunocompromised patients with hematological malignancies^14–16^ or after allogeneic transplantation^17^ developing COVID-19 infection have unfavorable outcomes. Our results might indicate that young subjects with a distant history of hematological malignancy are more susceptible to be infected when in contact with patients, but develop efficient immune responses. However, the small number of subjects do not allow us to draw firm conclusions on this topic on the basis of these data. The same hypothesis could be made regarding our group of mildly immunodeficient subjects, but the type of disease or treatment was heterogeneous (mainly immunosuppressive therapy for autoimmune disease, but also leucopenia, splenectomy, IgA deficiency, …), preventing us from advancing any conclusion on this topic.

In our study, long-term follow-up identified an active or previous solid tumor (and not hematological malignancy) as a risk factor for loss of seropositivity. It remains to be confirmed in large cohorts of cancer patients that protection against SARS-Cov-2 infection may be less durable in such seropositive patients. Finally, in some analyses, smoking was associated with a lower SARS-CoV-2 seroprevalence, which has also been observed in a French cohort^18^. Interestingly, smoking has been reported to increase ACE2 receptor expression^19^, to decrease the humoral response to SARS-CoV-2 vaccines^20^, and to increase the risks of COVID-19-related hospitalization and death^21^.

The main limitation of our study is that the population is mainly composed of young healthcare workers, who may not be representative of the overall population. Indeed, seroprevalence in our cohort decreased slightly with age. However, SARS-CoV-2 seroprevalence was comparable in administrative hospital staff and university researchers without occupational exposure to infected patients. On the other hand, the low numbers of subjects with severe comorbidities or with chronic medications may have precluded the identification of additional predictive factors. Nevertheless, the strength of this study is the prospective collection of many subject characteristics, including health information, exposure to COVID-19 patients, and symptoms, in a large population with a long-term follow-up.

In conclusion, we demonstrate a higher prevalence and persistence of SARS-CoV-2 natural antibodies in healthcare workers in contact with patients and in those with more severe symptoms (higher COVID-19 score). Interestingly, while immunocompromised participants were at higher risk to be seropositive, they appeared to retain this natural immunity, unlike those on chronic medications or with a solid tumor. It remains to be demonstrated whether these antibodies remain similarly protective in all patients, and whether these results can be extrapolated to vaccine immunity. As the emergence of variants weakens the collective immune protection, these data are crucial to tailor protective measures in the fight against the pandemic, especially among exposed front-line caregivers.

## Methods

### Study design and participants

The design of this study is described in Huynen et al.^1^. In this population, we collected general information and personal health history. For previously symptomatic participants, we calculated an empiric COVID-19 score partly implemented at CHU of Liège to screen patients before admission during the first waves of the pandemic.

This study was approved by the CHU of Liège Ethics Committee under number 2020/117 and has been performed in accordance with the Declaration of Helsinki. An informed consent form was signed by each participant. Data were recorded in a centralized database and pseudo-anonymized before statistical analysis.

### Serological testing

The method of serological testing is described in Huynen et al.^1^. In this part of the study, we only used the results of the IgG DiaSorin assay.

### Statistical analyses

Results are presented as means and standard deviation (SD) for continuous variables, and as frequency tables for qualitative variables, globally and for each serologic type. Univariate logistic regression models adjusted for time between March 1, 2020, and testing were applied to test seroprevalence with respect to demographics, clinical data, symptoms, and exposure. A multivariate logistic regression was done with stepwise selection. Results are reported as odds ratios (OR) and 95% confidence intervals. Results are considered significant at the 5% level (*p* < 0.05). Calculations were done using SAS version 9.4, and forest plots were generated using GraphPad Prism version 8.4.3.

## Supplementary Information

Supplementary Information.

## Data Availability

The study protocol and individual participant data that underlie the results reported in this article, after de-identification, can be shared with investigators whose proposed use of the data has been approved by the ethic committee of the University Hospital of Liège. Data can be provided for meta-analysis or other projects comparing the seroprevalence estimates in different regions. Requests should be addressed to the senior author at yves.beguin@chuliege.be.
