# Supplementary material for: Predictive factors for the presence and long-term persistence of SARS-CoV-2 antibodies in healthcare and university workers
Source: Sci Rep. 2022 Jun 13;12:9790. doi: 10.1038/s41598-022-13450-4 (PMC9191528; doi:10.1038/s41598-022-13450-4)
Supplement: Supplementary file 1 — Supplementary Information. [file 41598_2022_13450_MOESM1_ESM.docx]

**Predictive factors for the presence and long-term persistence of SARS-CoV-2 antibodies in healthcare and university workers**

Céline Grégoire^1¶^*, Pascale Huynen^2,3¶^, Stéphanie Gofflot^4^, Laurence Seidel^5^, Nathalie Maes^5^, Laura Vranken^6^, Sandra Delcour^6^, Michel Moutschen^7,8^, Marie-Pierre Hayette^2,3^, Philippe Kolh^9,10^, Pierrette Melin^2,3^, Yves Beguin^1,4,11^.

^1^ Division of Hematology, CHU of Liège, Liège, Belgium

^2^ Division of Medical Microbiology, Unilab, CHU of Liège, Liège, Belgium

^3^ Center for Interdisciplinary Research on Medicines, University of Liège, Liège, Belgium

^4^ Biothèque Hospitalo-Universitaire de Liège (BHUL), CHU of Liège, Liège, Belgium

^5^ Department of Biostatistics and Medico-economic Information, CHU of Liège, Liège, Belgium

^6^ Unilab, CHU of Liège, Liège, Belgium

^7^ Division of Infectious Diseases and General Internal Medicine, CHU of Liège, Liège, Belgium

^8^ GIGA-I3 Laboratory of Immunology, University of Liège, Liège, Belgium

^9^ Department of Information System Management, CHU of Liège, Liège, Belgium

^10^ Department of Biomedical and Preclinical Sciences, University of Liège, Liège, Belgium

^11^ GIGA-I3 Laboratory of Hematology, University of Liège, Liège, Belgium

^¶^ These authors contributed equally.
* Corresponding author

**Corresponding author:**

Grégoire Céline

Department of Hematology, CHU Sart-Tilman, Avenue de l’hôpital 1, 4000 Liège – Belgium

Tel +32 - 4 - 366 72 01; Fax +32 - 4 - 366 88 55

E-mail: [celine.gregoire@chuliege.be](mailto:celine.gregoire@chuliege.be)

**Supplementary Table S1.** Predictors of SARS-Cov-2 positive serology (IgG DiaSorin) in 3719 subjects in phase 1 (57 subjects did not consent to sharing personal health data): medical history and medications.

| **Predictive factor** | | **N (%)** | **Prevalence** | **OR (95% CI)** | | **P value** |
| --- | --- | --- | --- | --- | --- | --- |
| Diabetes mellitus | No | 3,615 (97.2) | 317 (8.8%) |  | |  |
|  | Yes | 104 (2.8) | 11 (10.6%) | 1.28 (0.67-2.42) | | 0.45 |
| Insulin | No | 78 (75) | 7 (9.0%) |  | |  |
|  | Yes | 26 (25) | 4 (15.4%) | 1.86 (0.50-6.97) | | 0.36 |
| Hypertension | No | 3,417 (91.2) | 297 (8.7%) |  | |  |
|  | Yes | 302 (8.1) | 31 (10.3%) | 1.16 (0.79-1.72) | | 0.45 |
| Heart disease | No | 3,696 (99.4) | 327 (8.8%) |  | |  |
|  | Yes | 23 (0.6) | 1 (4.3%) | 0.47 (0.06-3.49) | | 0.46 |
| Stroke | No | 3,701 (99.5) | 326 (8.8%) |  | |  |
|  | Yes | 18 (0.5) | 2 (11.1%) | 1.30 (0.30-5.72) | | 0.73 |
| Liver disease | No | 3,715 (99.9) | 327 (8.8%) |  | |  |
|  | Yes | 4 (0.1) | 1 (25%) | 2.95 (0.30-28.8) | | 0.35 |
| Kidney disease | No | 3,109 (99.7) | 328 (8.8%) |  | |  |
|  | Yes | 10 (0.3) | 0 (0.0%) | 0.41 (0.02-8.13) | | 0.56 |
| COPD | No | 3,688 (99.2) | 325 (8.8%) |  | |  |
|  | Yes | 31 (0.8) | 3 (9.7%) | 1.24 (0.37-4.13) | | 0.73 |
| Asthma | No | 3,376 (90.1) | 294 (8.7%) |  | |  |
|  | Yes | 343 (9.2) | 34 (9.9%) | 1.19 (0.81-1.73) | | 0.38 |
| Auto-immune disease | No | 3,466 (93.2) | 312 (9.0%) |  | |  |
|  | Yes | 253 (6.8) | 16 (6.3%) | 0.69 (0.41-1.16) | | 0.16 |
| Immunodeficiency | No | 3,700 (99.5) | 323 (8.7%) |  | |  |
|  | Yes | 19 (0.5) | 5 (26.3%) | 3.28 (1.16-9.26) | | 0.02 |
| Hematological malignancy | No | 3,703 (99.6) | 323 (8.7%) |  | |  |
|  | Yes | 16 (0.4) | 5 (31.3%) | 4.83 (1.65-14.1) | | 0.004 |
| Solid tumor | No | 3,611 (97.1) | 321 (8.9%) |  | |  |
|  | Yes | 108 (2.9) | 7 (6.5%) | 0.77 (0.35-1.67) | | 0.51 |
| General anesthesia since Feb 1 | No | 3,684 (99.1) | 325 (8.8%) |  | |  |
|  | Yes | 35 (0.9) | 3 (8.6%) | 0.96 (0.29-3.16) | | 0.94 |
| Ongoing pregnancy | No | 2,756 (99.2) | 239 (8.7%) |  | |  |
|  | Yes | 21 (0.8) | 2 (9.5%) | 1.01 (0.23-4.40) | | 0.99 |
| Medications | No | 1,407 (37.8) | 128 (9.1%) | |  |  |
|  | Yes | 2312 (62.2) | 200 (8.7%) | | 0.97 (0.76-1.22) | 0.76 |
| ACE inhibitor | No | 3,585 (96.4) | 315 (8.8%) | |  |  |
|  | Yes | 134 (3.6) | 13 (9.7%) | | 1.13 (0.63-2.04) | 0.68 |
| Sartan | No | 3,631 (97.6) | 321 (8.8%) | |  |  |
|  | Yes | 88 (2.4) | 7 (8.0%) | | 0.84 (0.38-1.83) | 0.65 |
| Statin | No | 3,619 (97.3) | 323 (8.9%) | |  |  |
|  | Yes | 100 (2.7) | 5 (5.0%) | | 0.54 (0.22-1.33) | 0.18 |
| Systemic corticosteroid | No | 3,705 (99.6) | 327 (8.8%) | |  |  |
|  | Yes | 14 (0.4) | 1 (7.1%) | | 0.80 (0.10-6.20) | 0.83 |
| Inhaled corticosteroid | No | 3,537 (95.1) | 312 (8.8%) | |  |  |
|  | Yes | 182 (4.9) | 16 (8.8%) | | 1.04 (0.61-1.77) | 0.88 |
| Oral contraceptive | No | 2,944 (79.2) | 262 (8.9%) | |  |  |
|  | Yes | 775 (20.9) | 66 (8.5%) | | 0.98 (0.74-1.30) | 0.86 |
| Proton-pump inhibitor | No | 3,441 (92.5) | 305 (8.9%) | |  |  |
|  | Yes | 278 (7.5) | 23 (8.3%) | | 0.94 (0.60-1.47) | 0.79 |
| Antihistaminic or antileukotriene | |  |  | |  |  |
|  | No | 3,333 (89.6) | 297 (8.9%) | |  |  |
|  | Yes | 386 (10.4) | 31 (8.0%) | | 0.89 (0.60-1.31) | 0.54 |
| Thyroid hormone | No | 3,057 (82.2) | 268 (8.8%) | |  |  |
|  | Yes | 662 (17.8) | 60 (9.1%) | | 1.05 (0.78-1.41) | 0.75 |
| Metformin | No | 3,534 (95.0) | 315 (8.9%) | |  |  |
|  | Yes | 185 (5.0) | 13 (7.0%) | | 0.81 (0.46-1.45) | 0.48 |
| Neurological drugs | No | 3,409 (91.2) | 303 (8.9%) | |  |  |
|  | Yes | 310 (8.3) | 25 (8.1%) | | 0.89 (0.58-1.37) | 0.61 |
| NSAID | No | 3,662 (98.5) | 326 (8.9%) | |  |  |
|  | Yes | 57 (1.5) | 2 (3.5%) | | 0.36 (0.09-1.48) | 0.15 |
| Immunosuppressive drugs | No | 3,661 (98.4) | 323 (8.8%) | |  |  |
|  | Yes | 58 (1.6) | 5 (8.6%) | | 0.90 (0.36-2.28) | 0.82 |

NSAID : nonsteroidal anti-inflammatory drug; COPD = chronic obstructive pulmonary disease; ACE = angiotensin converting enzyme; OR = odds ratio of univariate logistic regression models adjusted for time between March 1 and testing; CI = confidence interval.

**Supplementary Table S2.** Predictors of SARS-Cov-2 positive serology (IgG DiaSorin) in phase 1: detailed function.

| **Predictive factor** | **N (%)** | **Prevalence (%)** | **OR (95% CI)** | **P value** |
| --- | --- | --- | --- | --- |
| Secretary | 240 (6.4) | 11 (4.6) | - | 0.35 |
| Other administrative staff |  |  |  |  |
| Reception staff member | 72 (1.9) | 9 (12.5) | 3.45 (1.36-8.77) |  |
| Administrative staff member | 165 (4.4) | 14 (8.5) | 1.87 (0.83-4.25) |  |
| Technical staff |  |  |  |  |
| Porter | 39 (1.0) | 6 (15.4) | 3.28 (1.13-9.51) |  |
| Security guard | 12 (0.3) | 1 (8.3) | 2.18 (0.25-18.65) |  |
| Logistic assistant^a^ | 95 (2.5) | 7 (7.4) | 1.66 (0.62-4.43) |  |
| Cleaner | 163 (4.3) | 10 (6.1) | 1.29 (0.53-3.12) |  |
| Kitchen staff member | 54 (1.4) | 3 (5.6) | 0.92 (0.25-3.42) |  |
| Other technical staff member | 67 (1.8) | 3 (4.5) | 0.93 (0.25-3.44) |  |
| Paramedical staff |  |  |  |  |
| Nurse assistant | 31 (0.8) | 6 (19.4) | 4.04 (1.37-11.94) |  |
| Occupational therapist | 30 (0.8) | 5 (16.7) | 3.37 (1.08-10.55) |  |
| Speech therapist | 19 (0.5) | 2 (10.5) | 2.12 (0.43-10.39) |  |
| Nutritionist | 31 (0.8) | 2 (6.5) | 1.71 (0.36-8.14) |  |
| Physiotherapist | 90 (2.4) | 7 (7.8) | 1.61 (0.60-4.29) |  |
| Medical technologist^b^ | 70 (1.9) | 5 (7.1) | 1.53 (0.51-4.57) |  |
| Pharmacist | 75 (2.0) | 5 (6.7) | 1.49 (0.50-4.44) |  |
| Psychologist | 41 (1.1) | 2 (4.9) | 0.98 (0.21-4.61) |  |
| Social worker | 15 (0.4) | 0 (0.0) | - |  |
| Audiologist | 10 (0.3) | 0 (0.0) | - |  |
| Data manager | 34 (0.9) | 0 (0) | - |  |
| Laboratory staff |  |  |  |  |
| Laboratory technologist^c^ | 235 (6.2) | 20 (8.5) | 2.12 (0.99-4.55) |  |
| Other laboratory staff member | 48 (1.3) | 2 (4.2) | 0.94 (0.20-4.39) |  |
| Physician |  |  |  |  |
| Physician in training | 81 (2.1) | 11 (13.6) | 3.29 (1.36-7.95) |  |
| Physician | 601 (15.9) | 54 (9.0) | 2.14 (1.10-4.17) |  |
| Dentist | 45 (1.2) | 3 (6.7) | 1.39 (0.37-5.20) |  |
| Nurse | 1233 (32.7) | 131 (10.6) | 2.53 (1.34-4.77) |  |
| Researcher | 180 (4.8) | 17 (9.4) | 2.12 (0.97-4.66) |  |

^a^ In charge of stock management, transport of equipment, drugs and biological samples, distribution of meals to patients, ...

^b^ Responsible for conducting paramedical tests

**^c^** Manipulating biological samples

**Supplementary Table S3.** Predictors of SARS-Cov-2 positive serology (IgG DiaSorin) in all phases: subject characteristics.

| **Predictive factor** | | **N (%)** | **Mean ± SD (range)** | **Prevalence** | **OR (95% CI)** | **P value** |
| --- | --- | --- | --- | --- | --- | --- |
| Overall |  | 3289 | - | 404 (12.3%) | - | - |
| Sex | Female | 2,492 (75.9%) | - | 304 (12.2%) | - |  |
|  | Male | 790 (24.1%) | - | 100 (12.7%) | 1.06 (0.83-1.35) | 0.65 |
| Age | (year) | 3,282 | 41.6 ± 11.7  (21.5-81.3) |  | 0.989 (0.980-0.998) | 0.013 |
| Weight | (kilogram) | 3,232 | 70.0 ± 14.2  (33.0-150) |  | 1.006 (0.999-1.013) | 0.104 |
| Height | (centimeter) | 3,232 | 169 ± 8.80 (125-200) |  | 1.011 (1.000-1.023) | 0.061 |
| BMI | (kg/m²) | 3,232 | 24.5 ± 4.34  (15.6-55.1) | - | 1.010 (0.986-1.034) | 0.42 |
| Smoking | No | 2,816 (87.1%) | - | 354 (12.6%) | - |  |
|  | Yes | 416 (12.9%) | - | 41 (9.9%) | 0.74 (0.52-1.04) | 0.081 |

Place of residence, specific workplace, number/country/period of travel abroad, and type of mask used were also tested and found not significant. SD: standard deviation; BMI = body mass index; OR = odds ratio of univariate logistic regression models adjusted for time between March 1 and testing; CI = confidence interval.

**Supplementary Table S4.** Predictors of SARS-Cov-2 positive serology (IgG DiaSorin) in all phases: direct exposure.

| **Predictive factor** | | **N (%)** | **Prevalence (%)** | **OR (95% CI)** | **P value** |
| --- | --- | --- | --- | --- | --- |
| Time | Weeks from March 1 | 3282 |  | 1.030 (1.016-1.044) | <0.0001 |
| Function | Administrative staff | 441 (13.4%) | 46 (10.7%) | - | 0.024 |
|  | Researcher | 186 (5.70%) | 23 (12.4%) | 1.19 (0.70-2.02) |  |
|  | Laboratory staff | 220 (6.70%) | 23 (10.4%) | 1.04 (0.61-1.76) |  |
|  | Technical staff | 349 (10.6%) | 36 (10.3%) | 0.93 (0.58-1.47) |  |
|  | Paramedical staff | 418 (12.7%) | 39 (9.3%) | 0.82 (0.53-1.29) |  |
|  | Nurse | 1,087 (33.1%) | 158 (14.5%) | 1.45 (1.03-2.06) |  |
|  | Physician | 581 (10.6%) | 66 (12.6%) | 1.34 (0.91-1.98) |  |
| Patient contact | |  |  |  |  |
|  | No | 1,008 (30.7%) | 104 (10.3%) | - |  |
|  | Yes | 2,274 (69.3%) | 300 (13.2%) | 1.32 (1.05-1.68) | 0.020 |
| Handling of respiratory specimens | |  |  |  |  |
|  | No | 3,199 (97.5%) | 391 (12.2%) | - |  |
|  | Yes | 83 (2.50%) | 13 (15.7%) | 1.43 (0.78-2.61) | 0.25 |
| Contact with COVID patients | |  |  |  |  |
|  | No | 1,679 (51.3%)* | 157 (9.4%) | - |  |
|  | Yes | 1,596 (48.7%)* | 240 (15.0%) | 1.72 (1.39-2.14) | <0.0001 |
| Type of contact | |  |  |  |  |
|  | None | 1,679 (51.3%)* | 157 (9.4%) | - | <0.0001 |
|  | Familial | 60 (1.8%)* | 22 (36.7%) | 5.46 (3.14-9.50) |  |
|  | Occasional at work | 719 (22.0%)* | 91 (12.7%) | 1.42 (1.08-1.87) |  |
|  | Frequent/daily at work | 817 (24.9%)* | 127 (15.5%) | 1.79 (1.39-2.30) |  |
| COVID infection | |  |  |  |  |
|  | No | 3,149 (95.9%) | 317 (10.1%) | - |  |
|  | Yes | 133 (4.1%) | 87 (65.4%) | 16.1 (10.9-23.5) | <0.0001 |
| Method of diagnosis | |  |  |  |  |
|  | No infection | 3149 (95.9%) | 317 (10.1%) | - | <0.0001 |
|  | PCR diagnosis | 76 (1.3%) | 63 (82.9%) | 41.0 (22.2-76.0) |  |
|  | Clinical diagnosis | 57 (1.7%) | 24 (42.1%) | 6.23 (3.62-10.7) |  |

More detailed function (27 categories) was also tested: compared to secretaries, seroprevalence was significantly higher in reception staff members, administrative staff members, nurse assistants, stretcher bearer, researchers, dentist, occupational therapists, nurses, speech therapists, physicians, physicians in training and laboratory technologists. The other functions (laboratory staff member, logistic assistant, social worker, audiologist, kitchen staff member, data manager, nutritionist, physiotherapist, pharmacist, psychologist, technical staff member, cleaner, medical technician, security guard) were not identified as risk factors. OR = odds ratio of univariate logistic regression models adjusted for time between March 1 and testing; CI = confidence interval.

*7 missing

**Supplementary Table S5.** Predictors of SARS-Cov-2 positive serology (IgG DiaSorin) in 3239 subjects in all phases: medical history and medications.

| **Predictive factor** | | **N (%)** | **Prevalence (%)** | **OR (95% CI)** | **P value** |
| --- | --- | --- | --- | --- | --- |
| Diabetes mellitus | No | 3133 (96.9%) | 384 (12.3%) |  |  |
|  | Yes | 99 (3.10%) | 11 (11.1%) | 0.91 (0.48-1.72) | 0.77 |
| Insulin | No | 74 (74.7%) | 7 (9.46%) |  |  |
|  | Yes | 25 (25.3%) | 4 (16.0%) | 1.86 (0.49-7.00) | 0.36 |
| Hypertension | No | 2952 (91.3%) | 362 (12.3%) |  |  |
|  | Yes | 280 (8.70%) | 33 (11.8%) | 0.92 (0.63-1.35) | 0.68 |
| Heart disease | No | 3209 (99.3%) | 394 (12.3%) |  |  |
|  | Yes | 23 (0.70%) | 1 (4.35%) | 0.32 (0.043-2.37) | 0.26 |
| Stroke | No | 3215 (99.5%) | 393 (12.2%) |  |  |
|  | Yes | 17 (0.50%) | 2 (11.8%) | 0.95 (0.22-4.18) | 0.94 |
| Liver disease | No | 3229 (99.9%) | 394 (12.2%) |  |  |
|  | Yes | 3 (0.10%) | 1 (33.3%) | 3.36 (0.30-37.4) | 0.32 |
| Kidney disease | No | 3223 (99.7%) | 395 (12.3%) |  |  |
|  | Yes | 9 (0.30%) | 0 (0.0%) | 0.33 (0.016-6.72) | 0.47 |
| COPD | No | 3205 (99.2%) | 391 (12.2%) |  |  |
|  | Yes | 27 (0.80%) | 4 (14.8%) | 1.40 (0.48-4.08) | 0.55 |
| Asthma | No | 2924 (90.5%) | 358 (12.2%) |  |  |
|  | Yes | 308 (9.50%) | 37 (12.0%) | 0.999 (0.696-1.44) | 0.99 |
| Auto-immune disease | No | 2999 (92.8%) | 372 (12.4%) |  |  |
|  | Yes | 233 (7.20%) | 23 (9.87%) | 0.77 (0.50-1.21) | 0.26 |
| Immunodeficiency | No | 3215 (99.5%) | 389 (12.1%) |  |  |
|  | Yes | 17 (0.50%) | 6 (35.3%) | 3.64 (1.33-9.95) | 0.012 |
| Hematological malignancy | No | 3216 (99.5%) | 389 (12.1%) |  |  |
|  | Yes | 16 (0.50%) | 6 (37.5%) | 4.32 (1.55-12.0) | 0.0051 |
| Solid tumor | No | 3136 (97.0%) | 385 (12.3%) |  |  |
|  | Yes | 96 (3.00%) | 10 (10.4%) | 0.88 (0.45-1.71) | 0.70 |
| General anesthesia since Feb 1 | No | 3204 (99.1%) | 391 (12.2%) |  |  |
|  | Yes | 28 (0.90%) | 4 (14.3%) | 1.20 (0.41-3.48) | 0.74 |
| Ongoing pregnancy | No | 2437 (99.4%) | 295 (12.1%) |  |  |
|  | Yes | 15 (0.60%) | 2 (13.3%) | 1.08 (0.24-4.84) | 0.92 |
| Medications | No | 1158 (35.8%) | 153 (13.2%) |  |  |
|  | Yes | 2074 (64.2%) | 242 (11.7%) | 0.874 (0.703-1.086) | 0.22 |
| ACE inhibitor | No | 3109 (96.2%) | 381 (12.3%) |  |  |
|  | Yes | 123 (3.80%) | 14 (11.4%) | 0.919 (0.521-1.622) | 0.77 |
| Sartan | No | 3147 (97.4%) | 388 (12.3%) |  |  |
|  | Yes | 85 (2.60%) | 7 (8.24%) | 0.603 (0.276-1.318) | 0.20 |
| Statin | No | 3138 (97.1%) | 389 (12.4%) |  |  |
|  | Yes | 94 (2.90%) | 6 (6.38%) | 0.48 (0.21-1.11) | 0.085 |
| Systemic corticosteroid | No | 3220 (99.6%) | 394 (12.2%) |  |  |
|  | Yes | 12 (0.40%) | 1 (8.33%) | 0.65 (0.083-5.03) | 0.68 |
| Inhaled corticosteroid | No | 3065 (94.8%) | 376 (12.3%) |  |  |
|  | Yes | 167 (5.20%) | 19 (11.4%) | 0.95 (0.58-1.55) | 0.83 |
| Oral contraceptive | No | 2537 (78.5%) | 313 (12.3%) |  |  |
|  | Yes | 695 (21.5%) | 82 (11.8%) | 0.96 (0.74-1.25) | 0.77 |
| Proton-pump inhibitor | No | 2985 (92.4%) | 362 (12.1%) |  |  |
|  | Yes | 247 (7.60%) | 33 (13.4%) | 1.14 (0.77-1.67) | 0.52 |
| Antihistaminic or antileukotriene | No | 2904 (89.9%) | 357 (12.3%) |  |  |
|  | Yes | 328 (10.1%) | 38 (11.6%) | 0.94 (0.66-1.34) | 0.72 |
| Thyroid hormone | No | 2626 (81.3%) | 320 (12.2%) |  |  |
|  | Yes | 606 (18.8%) | 75 (12.4%) | 1.02 (0.78-1.34) | 0.88 |
| Metformin | No | 3060 (94.7%) | 381 (12.5%) |  |  |
|  | Yes | 172 (5.30%) | 14 (8.14%) | 0.64 (0.36-1.11) | 0.11 |
| Neurological drugs | No | 2946 (91.2%) | 366 (12.4%) |  |  |
|  | Yes | 286 (8.80%) | 29 (10.1%) | 0.79 (0.53-1.17) | 0.24 |
| NSAID | No | 3183 (98.5%) | 393 (12.4%) |  |  |
|  | Yes | 49 (1.50%) | 2 (4.08%) | 0.29 (0.071-1.21) | 0.090 |
| Immunosuppressive drugs | No | 3179 (98.4%) | 387 (12.2%) |  |  |
|  | Yes | 53 (1.60%) | 8 (15.1%) | 1.20 (0.56-2.58) | 0.63 |

NSAID : nonsteroidal anti-inflammatory drug; COPD = chronic obstructive pulmonary disease; ACE = angiotensin converting enzyme; OR = odds ratio of univariate logistic regression models adjusted for time between March 1 and testing; CI = confidence interval.

**Supplementary Table S6. Predictors of SARS-Cov-2 positive serology (IgG DiaSorin)** **in all phases: symptoms.**

| **Predictive factor** | | | **N (%) *** | **Prevalence (%)** | **OR (95% CI)** | **P value** |
| --- | --- | --- | --- | --- | --- | --- |
| Symptoms before testing  No | | | 2516 (76.8) | 224 (8.9) | - |  |
|  | Yes | | 759 (23.2) | 216 (28.5) | 4.34 (3.48-5.40) | <0.0001 |
| Covid score* | | 1-3 | 313 (9.56) | 31 (9.9) | 1.18 (0.77-1.80) | <0.0001 |
|  | | 4-10 | 338 (10.3) | 122 (36.1) | 5.86 (4.47-7.69) |  |
|  | | 11-17 | 108 (3.3) | 63 (58.3) | 15.46 (10.2-23.3) |  |
| Cough | | No | 3003 (91.7) | 337 (11.2) | - |  |
|  | | Yes | 272 (8.3) | 103 (37.87) | 4.90 (3.71-6.47) | <0.0001 |
| Dyspnea | | No | 3165 (96.6) | 399 (12.6) | - |  |
|  | | Yes | 110 (3.4) | 41 (37.3) | 4.34 (2.89-6.52) | <0.0001 |
| Anosmia | | No | 3152 (96.2) | 343 (10.9) | - |  |
|  | | Yes | 123 (3.8) | 97 (78.9) | 26.6 (17.4-40.8) | <0.0001 |
| Ageusia | | No | 3157 (96.4) | 352 (11.2) | - |  |
|  | | Yes | 118 (3.6) | 88 (74.6) | 21.6 (14.2-32.8) | <0.0001 |
| Nasal symptoms | | No | 2990 (91.3) | 350 (11.7) | - |  |
|  | | Yes | 285 (8.7) | 90 (31.6) | 3.69 (2.78-4.89) | <0.0001 |
| Sore throat | | No | 3029 (92.5) | 391 (12.9) | - |  |
|  | | Yes | 246 (7.5) | 49 (19.9) | 1.80 (1.28-2.53) | 0.0007 |
| Abdominal pain | | No | 3168 (96.7) | 411 (13.0) | - |  |
|  | | Yes | 107 (3.3) | 29 (27.1) | 2.36 (1.49-3.72) | 0.0002 |
| Diarrhea | | No | 3105 (94.8) | 390 (12.6) | - |  |
|  | | Yes | 170 (5.2) | 50 (29.4) | 3.20 (2.25-4.56) | <0.0001 |
| Vomiting | | No | 3253 (99.3) | 434 (13.3) | - |  |
|  | | Yes | 22 (0.7) | 6 (27.3) | 2.79 (1.08-7.21) | 0.034 |
| Myalgia | | No | 3017 (92.1) | 325 (10.8) | - |  |
|  | | Yes | 258 (7.9) | 115 (44.6) | 6.75 (5.12-8.92) | <0.0001 |
| Headaches | | No | 2888 (88.2) | 311 (10.8) | - |  |
|  | | Yes | 387 (11.8) | 129 (33.3) | 4.24 (3.30-5.44) | <0.0001 |
| Fever | | No | 3114 (95.1) | 360 (11.6) | - |  |
|  | | Yes | 161 (4.9) | 80 (49.7) | 8.10 (5.80-11.3) | <0.0001 |

OR = odds ratio of univariate logistic regression models adjusted for time between March 1 and testing; CI = confidence interval. *COVID-19 score: cough or dyspnea = 4 points; anosmia or ageusia = 4 points; nasal symptoms or sore throat = 1 point; abdominal pain, diarrhea or vomiting = 1 point; myalgia = 1 point; headaches = 2 points; fever < 38°C = 1 point or fever ≥ 38°C = 4 points.

*14 missing

**Supplementary Figure S1. Predictors of SARS-Cov-2 positive serology (IgG DiaSorin) in phases 1, 2 and 3, in multivariate analyses.**

**a.** Binary model with subject characteristics, exposure and detailed symptoms

**b.** Binary model with subject characteristics, exposure and COVID-19 score
